# Long-term Follow-up and Histological Correlation of Peripheral Nervous System Alterations in Neurofibromatosis Type 2

**DOI:** 10.1007/s00062-021-01102-5

**Published:** 2021-10-15

**Authors:** Tim Godel, Philipp Bäumer, Said Farschtschi, Klaus Püschel, Barbara Hofstadler, Sabine Heiland, Mathias Gelderblom, Martin Bendszus, Christian Hagel, Victor-Felix Mautner

**Affiliations:** 1 Department of Neuroradiology, Neurological University Clinic, Heidelberg University Hospital, Im Neuenheimer Feld 400, 69120 Heidelberg, Germany; 2 Center for Radiology dia.log, Vinzenz-von-Paul Str. 8, 84503 Altötting, Germany; 3 Department of Neurology, University Medical Center Hamburg-Eppendorf, Martinistraße 52, 20246 Hamburg, Germany; 4 Department of Legal Medicine, University Medical Center Hamburg-Eppendorf, Martinistraße 52, 20246 Hamburg, Germany; 5 Institute of Neuropathology, University Medical Center Hamburg-Eppendorf, Martinistraße 52, 20246 Hamburg, Germany

**Keywords:** Magnetic resonance neurography, Dorsal root ganglia hyperplasia, Peripheral nerve lesions, Neurocutaneous syndrome, Polyneuropathy

## Abstract

**Purpose:**

To examine long-term alterations of the dorsal root ganglia (DRG) and the peripheral nerve in patients with neurofibromatosis type 2 (NF2) by in vivo high-resolution magnetic resonance neurography (MRN) and their correlation to histology.

**Methods:**

In this prospective study the lumbosacral DRG, the right sciatic, tibial, and peroneal nerves were examined in 6 patients diagnosed with NF2 and associated polyneuropathy (PNP) by a standardized MRN protocol at 3 T. Volumes of DRG L3–S2 as well as peripheral nerve lesions were assessed and compared to follow-up examinations after 14–100 months. In one patient, imaging findings were further correlated to histology.

**Results:**

Follow-up MRN examination showed a non-significant increase of volume for the DRG L3: +0.41% (*p* = 0.10), L4: +22.41% (*p* = 0.23), L5: +3.38% (*p* = 0.09), S1: +10.63% (*p* = 0.05) and S2: +1.17% (*p* = 0.57). Likewise, peripheral nerve lesions were not significantly increased regarding size (2.18 mm^2^ vs. 2.15 mm^2^, *p* = 0.89) and number (9.00 vs. 9.33, *p* = 0.36). Histological analyses identified schwannomas as the major correlate of both DRG hyperplasia and peripheral nerve lesions. For peripheral nerve microlesions additionally clusters of onion-bulb formations were identified.

**Conclusion:**

Peripheral nervous system alterations seem to be constant or show only a minor increase in adult NF2. Thus, symptoms of PNP may not primarily attributed to the initial schwannoma growth but to secondary long-term processes, with symptoms only occurring if a certain threshold is exceeded. Histology identified grouped areas of Schwann cell proliferations as the correlate of DRG hyperplasia, while for peripheral nerve lesions different patterns could be found.

## Introduction

Neurofibromatosis type 2 (NF2) is an autosomal dominant inherited tumor syndrome characterized by the development of schwannomas as the predominant tumor entity [1]. Initially, a compressive impact of mass schwannomas on peripheral nerves was thought to be responsible for the development of NF2 associated polyneuropathy (PNP) [2]. More recent studies considered multiple Schwann cell tumorlets along peripheral nerves, radiological classified as peripheral nerve lesions, to be responsible for the development of PNP, with a close correlation between the number of noncompressive lesions and the severity of associated symptoms [3–6].

A further study using high-resolution in vivo magnetic resonance neurography (MRN) found distinct dorsal root ganglia (DRG) hyperplasia and identified primary sensory neurons as a possible vulnerable site of origin of areflexia and sensory loss in NF2. Besides bilateral vestibular schwannoma as the hallmark of NF2, DRG hyperplasia has further been confirmed as a highly accurate and easily investigable pathognomonic marker in the discrimination to schwannomatosis as the third neurofibromatosis subtype [7]. A subsequent MRN study that investigated children with NF2 revealed that both DRG hyperplasia and peripheral nerve microlesions occur to a similar extent compared to adults with severe PNP. These observations suggest that alterations of the peripheral nervous system (PNS) occur very early in the time course of NF2, show a limited instead of a linear or continuous growth, and additional long-term processes might be responsible for development of neuropathic symptoms.

To examine long-term alterations of the PNS in NF2 and its histological background, we investigated the peripheral nerve segments of the lower extremity by initial and follow-up examinations, and in one patient by ex vivo histology.

## Subjects and Methods

### Clinical and Demographic Patient Data

This study was performed in accordance with the Declaration of Helsinki, approved by the institutional ethics board (S398-2012) and written informed consent was obtained from all patients. Moreover, written informed consent for autopsy and post-mortem studies in one patient was obtained during lifetime. All patients were in neurological long-term care. Clinical examination, neurological and genetic testing was performed by a neurologist with more than 35 years of experience with NF2. Initial examinations were performed between 12/2009 and 5/2017 and follow-up examinations between 1/2014 and 6/2020. Overall, we included six patients with NF2 (five males, one female) and associated PNP (Table 1). All patients fulfilled the modified National Institutes of Health criteria for definite NF2 and diagnosis was further confirmed by genetic testing [8].

**Table 1:** Patient demographics and clinical data

Patient	Mutation	Gender/age in years (initial)	Follow-up (months)	PNP type and distribution	PNP grade (initial)	PNP grade (follow-up)	New clinical symptoms (initial—follow-up)	Mean DRG volume (L3–S2) (initial)	Mean DRG volume (L3–S2) (follow-up)	Peripheral nerve lesions (initial)	Peripheral nerve lesions (follow-up)
1	Sporadic (c.169 C > T;pArg57)	M, 23	99	Axonal-demyelinating, predominantly motor PNP of both legs. No sensory symptoms	++	++	–	447.03 mm^3^	595.81 mm^3^	Micro: 4 Intermediate: 2 Macro: 0	Micro: 4 Intermediate: 2 Macro: 0
2	Sporadic (c.448- G > A)	M, 23	33	Predominantly axonal, sensorimotor PNP, affecting both arms and both legs	+	++	–	551.83 mm^3^	615.37 mm^3^	Micro: 3 Intermediate: 5 Macro: 0	Micro: 2 Intermediate: 6 Macro: 0
3	Sporadic (c.1575-?_1737+?del)	M, 59	84	Predominantly axonal, predominantly motor PNP of both legs. No sensory symptoms	+	++	Ataxia	533.66 mm^3^	567.84 mm^3^	Micro: 9 Intermediate: 1 Macro: 0	Micro: 9 Intermediate: 1 Macro: 0
4	Sporadic (not detected in blood)	F, 58	100	Axonal, sensorimotor PNP, affecting both arms and both legs	+	+	–	181.30 mm^3^	183.93 mm^3^	Micro: 0 Intermediate: 2 Macro: 0	Micro: 2 Intermediate: 2 Macro: 0
5	Sporadic (deletion Exon 1)	M, 20	96	Axonal, sensorimotor PNP, affecting both legs	+++	+++	Progressive quadriceps paresis	1432.15 mm^3^	1546.42 mm^3^	Micro: 5 Intermediate: 4 Macro: 0	Micro: 6 Intermediate: 3 Macro: 0
6	Sporadic (not detected in blood)	M, 41	14	Axonal-demyelinating, sensorimotor PNP, affecting both legs and the left arm	++	+++	Singultus, vomiting, death	6006.88 mm^3^	6215.50 mm^3^	Micro: 10 Intermediate: 7 Macro: 2	Micro: 10 Intermediate: 7 Macro: 2

Classification of clinical PNP severity grade: + subclinical, ++ moderate, +++ severe.

*DRG* Dorsal root ganglia, *PNP* Polyneuropathy

### Electrophysiological Testing

Initial and follow-up nerve conduction studies and electromyography were performed by board-certified neurologists using a standardized protocol (Table 1). Motor nerve conduction studies included measurement of compound muscle action potential, distal motor latencies, conduction velocities in the lower leg, F‑wave latencies, and presence of the tibial and peroneal nerve of both sides. Sensory nerve conduction studies of both sural nerves included the amplitude of compound sensory nerve action potential, and conduction velocity (antidrome technique/surface electrodes). The performance of needle electromyography of selected muscles was guided by clinical suspicion of muscle involvement.

### Classification of Clinical Severity of NF2-PNP

Electrophysiological results were categorized according to following criteria into subclinical, moderate and severe and are given in Table 1:

- Subclinical: nerve conduction and electromyography evidence of axonal loss and/or demyelination in the distribution of at least two peripheral nerves.
- Moderate: absent ankle jerks, slight symmetrical hypesthesia, and paresis of distal muscle groups in addition to electrophysiological signs of PNP.
- Severe: additional physical findings that indicated severe motor weakness, atrophy of muscles, deformities of hands or feet, analgesia and loss of sensory modalities in the distribution of at least two peripheral nerves.

### Imaging Protocol

Initial and follow-up MRN examinations were conducted on 3 T magnetic resonance scanners (Magnetom SKYRA, Magnetom VERIO and Magnetom TIM TRIO, Siemens Healthineers, Erlangen, Germany). A 15-channel transmit/receive spine coil and an 8‑channel receive body flex coil (Siemens Healthcare) were used for imaging of the lumbosacral plexus, and a 15-channel transmit/receive knee-coil (Invivo, Gainesville, FL, USA) was used for imaging the lower extremity. All patients underwent large-coverage high-resolution imaging of the lumbosacral plexus and the right lower extremity including:

1. A 3D T2-weighted Sampling-Perfection-with-Application-optimized-Contrasts-using-different-flip-angle-Evolution (SPACE) Short-Tau-Inversion-Recovery (STIR) sequence of the lumbosacral plexus with the following parameters: effective echo time 68 ms, repetition time/echo time 3000/208 ms, inversion time 210 ms, field of view 305 × 305 mm^2^, slice thickness 0.95 mm, matrix size 320 × 320 × 104, no gap, voxel size 0.95 × 0.95 × 0.95 mm^3^, acquisition time 8:35 min, covering the lumbosacral spine from the 2nd lumbar vertebra to the coccyx. Moreover, 3D reformations were acquired in axial, sagittal and coronal orientations.
2. Large coverage, T2-weighted, turbo-spin-echo sequences of the right thigh, knee and calf level in an axial orientation, using the following parameters: echo time 59 ms, repetition time 8.470 ms, spectral fat saturation, matrix 512 × 512, field of view 160 × 160 mm, voxel size 0.3 × 0.3 × 3.5 mm^3^, interslice gap 0.35 mm, 45 slices for each slab, acquisition time 7:56 min per slab.

### Imaging Analysis

**Fig. 1:**
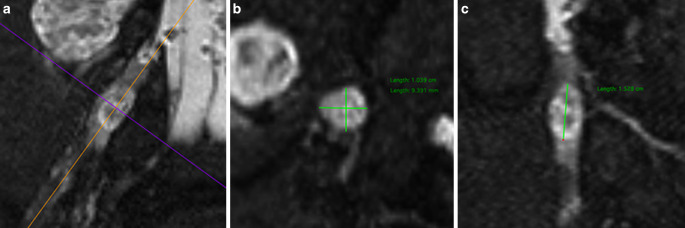
Measurement of DRG volume. DRG volume was assessed in the T2-weighted 3D-image by using the mathematic equation for volume assessment of an ellipsoid: volume in mm^3^ = π/6 * (A × B × C), where A represents the diameter in axial plane (**b**), B the diameter at 90° in the axial plane (**b**), and C the length in a reformation along the long axis of the DRG (**c**), which was determined individually of each DRG (**a**)

Image post-processing was performed by 2 independent raters with 3 and 7 years of experience in peripheral nerve imaging, who were blinded to each other. The DRG volume was assessed in the T2-weighted 3D image of the lumbosacral plexus by measuring the largest diameter of the DRG L3–S2 in axial, coronal, and sagittal reformations using Osirix (Pixmeo, Bernex, Switzerland). Volumes were calculated in accordance to the mathematic equation for volume assessment of an ellipsoid: volume in mm^3^ = π/6 * (A × B × C), where A represents the diameter in axial plane, B the diameter at 90° in the axial plane, and C the length in a reformation along the long axis of the DRG, which was determined individually of each DRG (Fig. 1). Assessment of fascicular T2-signal was performed in the initial and follow-up examinations within the right sciatic, tibial, and peroneal nerves slice-by-slice from proximal to distal. Peripheral nerve lesions were identified by an increased fascicular T2 signal and classified into one of the following categories by their largest cross-sectional diameter [4]:

1. microlesion if the largest diameter of the lesion was < 2 mm.
2. intermediate lesion if the largest diameter of the lesion was between 2 and 5 mm.
3. macrolesion if the largest diameter of the lesion was > 5 mm.

### Statistical Analysis

Statistical analyses were performed using GraphPad Prism 7.0 (GraphPad Software, La Jolla, CA, USA). Mean values of DRG volume, mean diameter of all peripheral nerve lesions and mean number of nerve lesions in each of the categories listed above were calculated in the initial and follow-up examinations. The results were tested for statistical significance using a paired, two-sided Student’s t‑tests, with the significance level set at *p* < 0.05. All results are documented as mean values ± standard deviation. Pearsonʼs r was reported as a measure of interrater reliability of DRG volume, amount and diameter of peripheral nerve lesions.

### Postmortem Studies and Histological Analyses

Patient 6 (Table 1) died on 6/2019 after multimodal long-term treatment of NF2-associated complications by respiratory paralysis in order to compression of the brainstem by incurable vestibular schwannomas. The patient was immediately transferred from the place of death to the Department of Legal Medicine and an autopsy was carried out the next day by a forensic pathologist with more than 40 years of experience.

For detailed analyses of the PNS, the brain was detached from the spinal cord at the level of the foramen magnum and the spinal canal was accessed from dorsally through resection of the vertebral arches. After the spinal cord was removed en bloc with the spinal nerves and DRG of the lumbosacral plexus, the right sciatic nerve was dissected from the greater sciatic foramen to its division into the tibial and peroneal nerve at the mid-knee level. Autopsy specimens were preserved in a formalin solution for further analyses. A neuropathologist with more than 30 years of experience performed detailed histological analysis. The investigations comprised hematoxylin and eosin (H&E) stained micrograph sections of spinal nerves and DRG of the lumbosacral plexus, as well as multiple sections of the right sciatic, tibial and peroneal nerves. Moreover, neurofilament (NF) stained micrograph sections were performed for the lumbosacral DRG and the sciatic, tibial and peroneal nerves. Additionally, peripheral nerve tumors were labeled with antibodies against epithelial membrane antigen, somatostatin receptor 2a, S100 protein, protein kinase C potentiated inhibitor, proliferation associated antigen Ki-67 and glial fibrillary acidic protein [9].

## Results

**Fig. 2:**
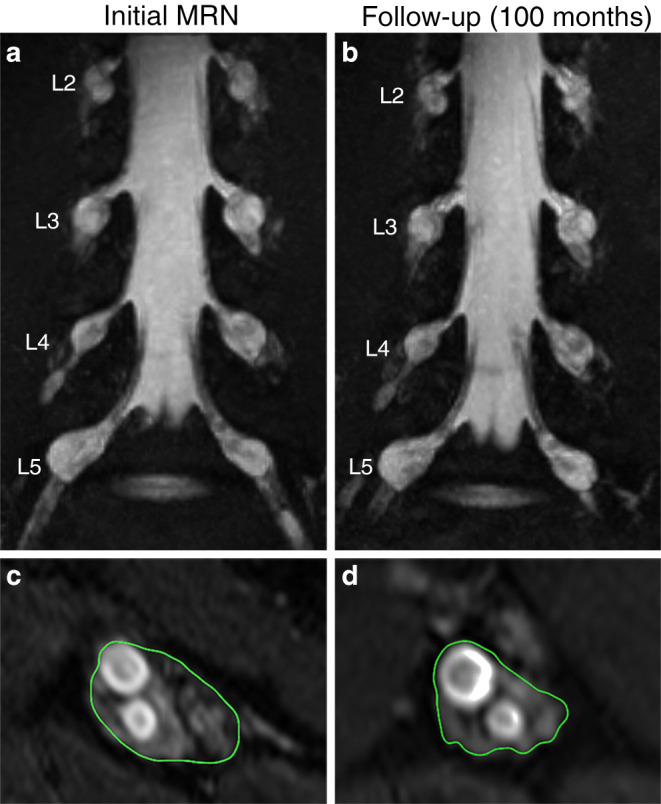
Dorsal root ganglia (DRG) volume and peripheral nerve lesions in the initial (**a**, **c**) and follow-up examination after 100 months (**b**, **d**). Coronal 15 mm maximum intensity projection volume rendering figure of the lumbosacral plexus (**a**, **b**) and an axial, T2w, fat-saturated sequence of the sciatic nerve at the thigh level (**c**, **d**). DRG hyperplasia as well as peripheral nerve lesion diameter and count have not changed significantly

Volumes of DRG L3–S2 as well as peripheral nerve lesion diameters and counts per category were quantitatively assessed for a total of 6 NF2 patients by initial and follow-up examinations 70.0 ± 37.7 months later (range 14–100 months) (Fig. 2, Table 1).

DRG volumes were not significantly increased in follow-up examinations by 0.41% for L3 (3267.61 ± 9802.03 mm^3^ vs. 3281.11 ± 9800.00 mm^3^, *p* = 0.10), 22.41% for L4 (654.69 ± 522.27 mm^3^ vs. 801.39 ± 875.84 mm^3^, *p* = 0.23), 3.38% for L5 (731.81 ± 498.15 mm^3^ vs. 756.56 ± 504.75 mm^3^, *p* = 0.09), 9.79% for S1 (2717.29 ± 4008.46 mm^3^ vs. 3006.04 ± 4316.99 mm^3^, *p* = 0.05), and 1.17% for S2 (255.97 ± 211.14 mm^3^ vs. 258.96 ± 214.63 mm^3^, *p* = 0.57). A high interrater reliability was found for the evaluation of DRG volumes, with Pearsonʼs r of 0.89 (initial and follow up examination).

Likewise, diameters of peripheral nerve lesions were not significantly alternated in follow-up MRN from 2.18 ± 1.09 mm to 2.15 ± 1.04 mm, *p* = 0.36. Peripheral nerve lesion count was not significantly increased in follow-up examinations for microlesions (5.17 ± 3.76 vs. 5.50 ± 3.45, *p* = 0.47), and unchanged for intermediate lesions (3.50 ± 2.26 vs. 3.50 ± 2.43, *p* = 1.00) and macrolesions (0.33 ± 0.82 vs. 0.33 ± 0.82, *p* = 1.00). A high interrater reliability was found for the subsequent evaluation of peripheral nerve lesions, with Pearsonʼs r of 0.91 for total lesion count and 0.95 for lesion diameter (initial and follow up examination).

### Histological Findings and the Correlation to MRN

**Fig. 3:**
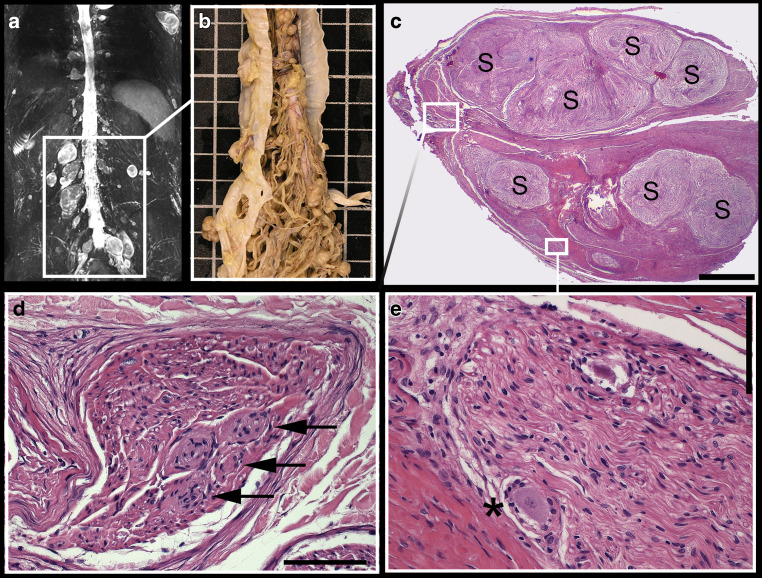
Dorsal root ganglia (DRG) hyperplasia in MR-Neurography, dissected spinal cord (**b**) and representative histological sections of the right DRG S1 (**c–e**). Imaging and section specimen of the spinal cord revealed a marked and diffuse enlargement of the lumbosacral DRG (**a**, **b**). Hematoxylin and eosin (H&E) staining revealed that DRG are massively infiltrated by schwannoma tissue (S) displacing the neuronal tissue to the periphery (**c**). Thus, the regular division in a central nerve fiber-rich area and a peripheral cell body-rich area is completely abrogated and only a very few, frequently degenerated sensory cell bodies could be detected (**e**, *asterisk*). Moreover, multiple onion-bulbs were observed within the region of the entering axons of the spinal nerve (**d**, *arrows*). Scale in **b** (*white squares*) = 10 mm, in **c** = 1 mm, in **d** = 100 µm, in **e** = 100 µm

In the cerebellar-pontine angle bilateral schwannomas of about 40 mm diameter with a subsequent tonsillar herniation were found as the cause of death. In position of the lumbosacral DRG L3–S2 on both sites, predominantly schwannoma tissue was found, encapsulated by a thick layer of connective tissue (Fig. 3). Detailed H&E analysis of the DRG L3–S2 revealed severe alterations within the internal organization with a subtotal loss of neuronal cell bodies and small sharply demarcated Schwann cell proliferations. The regular internal structure of the DRG, with neuronal cell bodies in the peripheral and nerve fibers in the central zone, was not delimitable. Instead, only very few sensory cell bodies were detected in the DRG’s periphery and completely surrounded by grouped schwannoma tissue. Similarly, the central zone of the DRG was completely permeated by multiple schwannomas. Additional NF staining revealed subperineural bundles of axons surrounded by multiple, predominantly coreless onion-bulb formations. There was no increased mitotic activity and no necrosis (schwannomas WHO grade I). Upon immunohistochemical investigation all schwannomas expressed S100-protein and protein kinase C potentiated inhibitor.

**Fig. 4:**
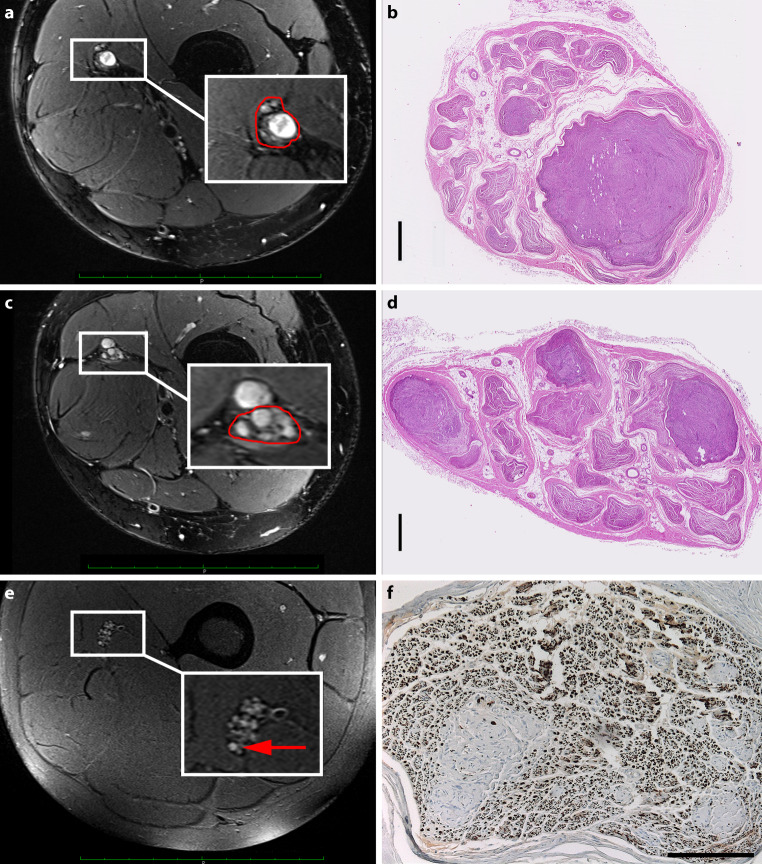
Typical imaging appearance of an intermediate lesion (**a**) and microlesions (**c**, **e**) in MR-Neurography and corresponding histological findings (**b**, **d**, **f**). Axial, T2w, fat-saturated sequence of the sciatic nerve at the thigh level, showing an intermediate lesion (**a**), several microlesions with fascicular hyperplasia (**c**) and a microlesion with normal fascicular diameter (**e**). Corresponding hematoxylin and eosin (H&E) stains of the sciatic nerve revealed a central schwannoma with peripherally displaced axon bundles as the underlying finding of the intermediate lesion (**b**) and several tiny schwannomas as the underlying histomorphologic correlate of microlesions with fascicular hyperplasia (**d**). Moreover, fascicular neurofilament (NF) stain shows multiple onion-bulb formations of proliferating Schwann cells with whorls around centrally located axons as a correlate of a microlesion with normal fascicular caliber (**f**). Scale in **a**, **c**, **e** = 10 cm, in **b, d** = 1 mm, in **f** = 200 µm

Detailed proximal to distal correlation of NF and H&E micrograph sections of the right sciatic nerve revealed central schwannomas with peripherally displaced axon bundles as the underlying findings of macrolesions and intermediate lesions (> 5 mm and 2–5 mm) detected in MRN (Fig. 4). For microlesions (< 2 mm), two different underlying patterns could be found. While focal fascicular hypertrophies matched with tiny intrafascicular schwannomas in H&E sections, fascicular T2-hyperintensities with normal fascicular diameter may be attributed to onion-bulb formations of proliferating Schwann cells forming whorls around centrally located axons in NF staining.

## Discussion

This study examined long-term alterations of the PNS in six patients with NF2 and associated PNP. A major finding of this study is that DRG hyperplasia as well as peripheral nerve lesions seems to be relatively constant or show only a minor increase in adults with NF2. This observation is supported by a previous study that examined the PNS of infants and adults with NF2 [10]. Although none of the examined children exhibited signs and symptoms suggestive for PNP, DRG hyperplasia and peripheral nerve lesions were already present to a similar extent compared to adult patients with long-time history of severe PNP. Thus, it has been hypothesized, if structural alterations of the PNS might develop very early in the time course of NF2, but show a limited instead of linear growth, as progression rates tend to decrease with increasing age. This observation is consistent with previous longitudinal studies, reporting decreased schwannoma growth rates of the VIII cranial nerve with increasing age [11–13]. Applicable explanations for this observation could be a reduced growth rate of somatic cells or the lower number of proliferating cells over time [12]. In this respect it does not seem remarkable that the dynamics of structural PNS alterations found in this study did not reach level of significance, even in long-term follow-up examinations up to 100 months after initial examination.

On the other hand, PNP in NF2 is typically absent in younger age and up to two thirds of patients become symptomatic during the later decades [5, 6]. To explain this marked discrepancy, secondary processes within the DRG and peripheral nerves have been considered to be responsible for the development of PNP in NF2. A previous imaging study demonstrated a positive correlation between the number of peripheral nerve microlesions and the severity of neuropathic symptoms [4]. As those nerve lesions are already evident in asymptomatic children, it could be hypothesized that primary changes of the T2-signal may reflect the pathophysiologically underlying Merlin deficiency, but that PNP and associated symptoms occurs if axonal sprouting and recruiting can no longer compensate neuronal dysfunction [14]. While solid Schwannomas in different diameters were identified as the histopathological correlate of macrolesions and intermediate lesions in MRN, for microlesions two different histological patterns could be found. First, microlesions associated with both an elevated T2 signal and also an increased fascicular diameter could be linked to tiny Schwann cell tumorlets that are thought to represent clusters of dysplastic Schwann cells. Second, microlesions solely with an increased T2 signal, but normal fascicular diameter correlated with areas of predominantly coreless onion-bulb formations. Thus, it could be assumed that non-hypertrophied peripheral nerve microlesions might primarily reflect Merlin dysfunction at a molecular level with corresponding Schwann cell dedifferentiation on a histological level. While these changes are not associated with symptoms yet, the subsequent formations of coreless onion-bulbs with degeneration of the central axon led to the axonal character of NF2-associated PNP [6].

Moreover, this study examined the histological background of DRG hyperplasia as a potential pathognomonic marker in human NF2. Structural alterations of the proximal peripheral nerve segment in NF2 were first described in the murine model as a generalized swelling of both the DRG and the proximal nerve roots [15]. Alterations of DRG morphology are also a very early feature of human NF2 that is already evident in children and infants [7, 10]. Histopathological analyses of the DRG in murine NF2 identified grouped Schwann cell hyperplasia as the underlying correlate of DRG hyperplasia [15]. This study confirmed grouped Schwann cell proliferates as the pathostructural correlate of DRG hyperplasia also in human NF2. In contrast to the murine model, sensory nerve cells bodies, which are physiologically located in the peripheral zone of the DRG, were almost completely absent and displaced by schwannoma tissue. It is well known that sensory nerve cells are susceptible to damage by compression, e.g. due to disc herniation [16]. Thus, chronically increasing pressure due to Schwann cell hyperplasia within the DRG may contribute to a progressive dysfunction of sensory cells over time and might lead to a subsequent degeneration of the nerve cell and its corresponding axon. Interestingly, patients 1 and 3 showed no sensory symptoms, but marked DRG hyperplasia with comparable hyperplasia rates to patients with sensory symptoms. Thus, it could be hypothesized that DRG enlargement does not necessarily cause sensory symptoms in all NF2 patients and sensory symptoms may be attributed to both DRG enlargement and peripheral nerve lesions.

The reason why grouped areas of Schwann cell proliferations arise nearby sensory neurons in NF2, but not in schwannomatosis, still remains obscure. A critical role could be attributed to satellite cells that are present as multipotent glia progenitor cells in the central nervous system (CNS) as well as in the PNS. Depending on the local environment these cells can differentiate into astrocytes and oligodendrocytes in the CNS or Schwann cells in the PNS [17, 18]. Since intraganglionic satellite cells are found in sensory ganglia of cranial and spinal nerves, it could be speculated that hyperplasia of those cells might lead to both development of Schwann cell hyperplasia within sensory ganglia of cranial and spinal nerves. According to this hypothesis, bilateral vestibular schwannoma might only represent a very local manifestation of a generalized sensory ganglia hyperplasia syndrome. While sensory ganglia of the spine are embedded in loose connective tissue in general, the limited space within the internal bony auditory canal might contribute to an early increased compression of ganglia cells and explain the early onset of auditory and vestibular symptoms as opposed to the late onset of PNS symptoms.

This study comes along with limitations. Although all examined patients exhibited slightly increased DRG volume over time (especially patients 1 and 2), this did not reach a significant level. This could also be attributed to the small sample size of patients in this study. Second, DRG volumes in this study were estimated by using the equation V = π/6 * A * B * C, which assumes an exact ellipsoid shape as a structure for precise volume calculation. Especially in grossly remodeled DRGs, this exact ellipsoid shape may not be given and the absolute calculated volume may be overestimated or underestimated, while the percentage ratio of volume alteration between the initial and follow-up examination may not be affected. Third, the assumption that gross alterations of the PNS in NF2 take place in early childhood and are restricted later on, are somewhat hypothetical, as this should be evaluated in long-term longitudinal studies, starting at birth or very early childhood and following these children to adult ages.

## Conclusion

In conclusion, DRG hyperplasia and peripheral nerve lesion seem to be relatively constant or show only a minor increase in adult NF2. Thus, symptoms of PNP may not primarily be attributed to the initial schwannoma growth but to secondary long-term processes, with symptoms only occurring if a certain threshold is exceeded. Histological sections revealed grouped areas of Schwann cell proliferation as the structural correlate of DRG hyperplasia of human NF2. While solid schwannomas were identified as the histopathological correlate of macrolesions, intermediate and microlesions, for the latter, also Schwann cell tumorlets and coreless onion-bulb formations were identified.
